# Interconnections of Pseudomonas aeruginosa Quorum-Sensing Systems in Intestinal Permeability and Inflammation

**DOI:** 10.1128/mbio.03524-22

**Published:** 2023-02-14

**Authors:** Vijay K. Singh, Marianna Almpani, Kelsey M. Wheeler, Laurence G. Rahme

**Affiliations:** a Department of Surgery, Harvard Medical School and Massachusetts General Hospital, Boston, Massachusetts, USA; b Department of Microbiology, Harvard Medical School, Boston, Massachusetts, USA; c Shriners Hospitals for Children, Boston, Massachusetts, USA; Emory University School of Medicine

**Keywords:** *Pseudomonas aeruginosa*, quorum sensing, virulence, antivirulence, intestinal permeability, intestinal inflammation, MvfR, PqsR, LasR, RhlR

## Abstract

Quorum sensing (QS) is a highly conserved microbial communication mechanism based on the production and sensing of secreted signaling molecules. The recalcitrant pathogen Pseudomonas aeruginosa is a problematic nosocomial pathogen with complex interconnected QS systems controlling multiple virulence functions. The relevance of QS in P. aeruginosa pathogenesis is well established; however, the regulatory interrelationships of the three major QS systems, LasR/LasI, MvfR (PqsR)/PqsABCD, and RhlR/RhlI, have been studied primarily *in vitro*. It is, therefore, unclear how these relationships translate to the host environment during infection. Here, we use a collection of P. aeruginosa QS mutants of the three major QS systems to assess the interconnections and contributions in intestinal inflammation and barrier function *in vivo*. This work reveals that MvfR, not LasR or RhlR, promotes intestinal inflammation during infection. In contrast, we find that P. aeruginosa-driven murine intestinal permeability is controlled by an interconnected QS network involving all three regulators, with MvfR situated upstream of LasR and RhlR. This study demonstrates the importance of understanding the interrelationships of the QS systems during infection and provides critical insights for developing successful antivirulence strategies. Moreover, this work provides a framework to interrogate QS systems in physiologically relevant settings.

## OBSERVATION

Pseudomonas aeruginosa exemplifies clinically problematic pathogens with complex quorum-sensing (QS) networks. The P. aeruginosa QS systems control multiple acute and chronic functions contributing to severe systemic infections (1–15). At least three major interconnected QS systems function in P. aeruginosa under the control of specific regulators, LasR, MvfR, and RhlR (2, 5, 8, 14, 15). Elucidating the relationships between the QS systems in controlling the systemic consequences of infection is critical in designing successful antivirulence strategies, especially because P. aeruginosa often accumulates specific mutations during infection, particularly in *lasR.* Loss of LasR function is associated with disease progression, exacerbations, and increased inflammation (16, 17). It is currently unclear how the three QS systems work in concert to regulate virulence during infection or how *lasR* mutations might impact the QS systems’ interrelationships and, in turn, the efficacy of anti-QS therapies during infection.

P. aeruginosa infections are often associated with disrupted intestinal integrity (18–20), permitting bacteria and their products to translocate from the gut to the bloodstream. This may lead to gut-derived sepsis associated with higher mortality (21, 22). Thus, P. aeruginosa wound infections and the resulting intestinal barrier dysfunction are important clinical considerations. The QS regulator MvfR is involved in systemic consequences in the host, including increased intestinal permeability and inflammation (19). In turn, pharmacological inhibition of MvfR significantly alleviates bacterial-induced gut permeability, decreases tumor necrosis factor alpha (TNF-α) levels in both serum and ileum, decreases bacterial dissemination, and abolishes the production of the MvfR-regulated small molecules critical for acute and persistent infections (23).

Here, we aimed to interrogate the roles of the other two QS regulators, LasR and RhlR, and their interconnection to MvfR in P. aeruginosa dissemination and intestinal permeability and inflammation. Using double and triple QS mutants, we further dissected the P. aeruginosa QS systems’ interrelationships concerning these phenotypes.

**FIG 1:**
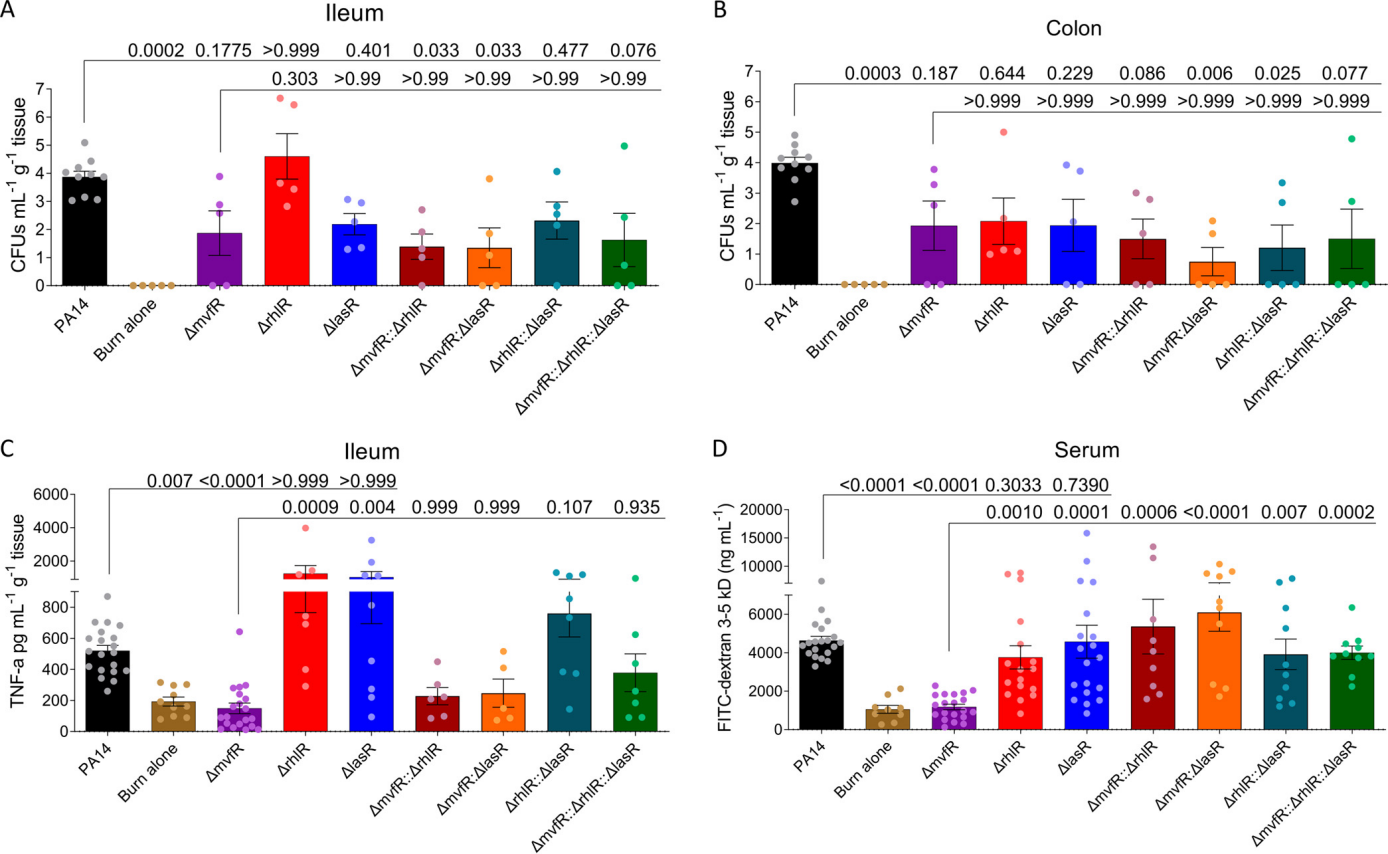
Dissemination of P. aeruginosa from the site of infection to the ileum (A) and colon (B). The inflammatory response in the ileum TNF-α levels (C) increased when mice were infected with Δ*lasR*, Δ*rhlR*, and WT strains, whereas the TNF-α level was similar to burn alone in Δ*mvfR*, double, and triple mutants. (D) The flow of the FITC-dextran from the intestinal lumen to the systemic circulation was not increased significantly in the Δ*mvfR* single mutant in comparison to burn alone, whereas the WT, Δ*lasR*, Δ*rhlR*, and double and triple mutants increased intestinal permeability. Each dot represents data from one mouse. The error bars denote ± SEM. Significance was assessed by Kruskal-Wallis nonparametric test with Dunnett's posttest applied. Exact *P* values are provided. A portion of the underlying data for the WT, burn alone, and Δ*mvfR* were previously published in reference 23. These data were reused and reanalyzed, as these experiments were performed in parallel to experiments with the other single, double, and triple mutants.

It has been reported that P. aeruginosa load in the ileum and colon was significantly reduced for the Δ*mvfR* strain relative to the wild-type (WT) strain (23). Here, we find that the double and triple mutants lacking *mvfR* were reduced in the ileum and colon relative to WT (Fig. 1A and B). In the ileum, only the Δ*rhlR* mutant was significantly more abundant than Δ*mvfR* (Fig. 1A). In the colon, none of the QS mutants were increased relative to Δ*mvfR* (Fig. 1B). Together, these findings suggest that each QS regulator is involved in dissemination or viability in the colon, although RhlR may be less important for dissemination or viability in the ileum.

While there were no significant differences between the Δ*mvfR* and Δ*lasR* in terms of their intestinal abundance, TNF-α levels were increased in the ileum by the Δ*lasR* relative to Δ*mvfR* (Fig. 1C). Double and triple mutants lacking a functional MvfR did not trigger elevated TNF-α levels relative to the Δ*mvfR* single mutant. The average TNF-α level was higher following infection with the double Δ*rhlR*::Δ*lasR* mutant relative to Δ*mvfR*, although the difference was not statistically significant (Fig. 1C). Taken together, these findings suggest that MvfR, but not LasR or RhlR, is involved in triggering the intestinal inflammatory response and that this role is independent of the ability of each mutant to disseminate to the ileum.

Mucosal inflammation is associated with increased intestinal permeability (24). Therefore, we next assessed how each QS regulator impacted intestinal permeability by measuring fluorescein isothiocyanate (FITC)-dextran 3- to 5-kDa flux from the intestinal lumen to the systemic circulation. While the Δ*mvfR* single mutant did not increase intestinal permeability relative to burn alone, the Δ*lasR* and Δ*rhlR* strains each increased intestinal permeability relative to Δ*mvfR* (Fig. 1D). Intriguingly, the double and triple mutants also increased intestinal permeability relative to Δ*mvfR* (Fig. 1D), indicating that LasR and RhlR may each have a role in maintaining intestinal barrier integrity that is downstream of MvfR.

**FIG 2:**
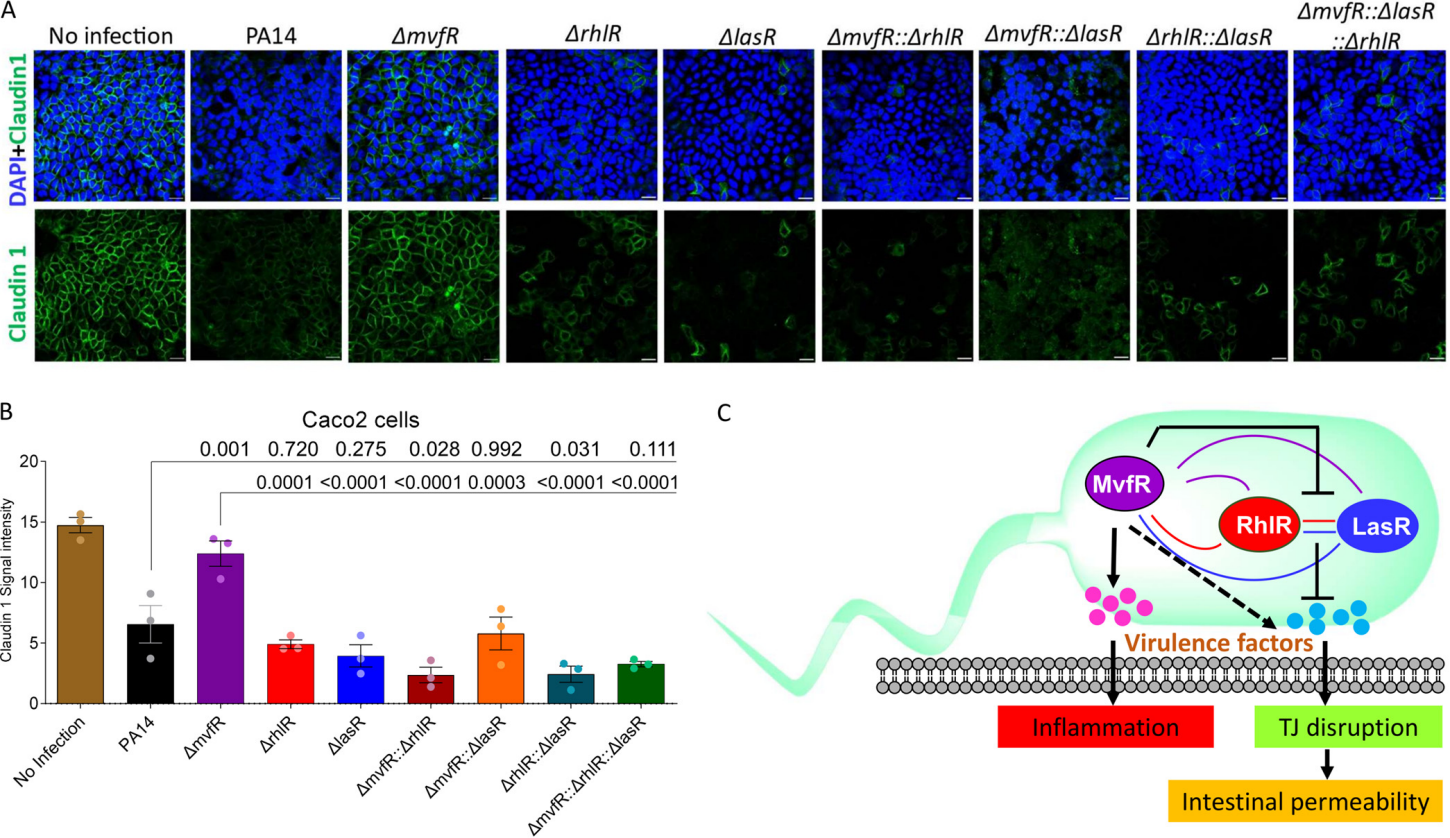
Immunofluorescence staining of the tight junction Caco-2 cells. (A) The integrity of the tight junction protein claudin-1 was compromised when cells were infected with WT, Δ*lasR*, Δ*rhlR*, and double and triple mutants, whereas it was unaffected in the cells infected with Δ*mvfR* mutants. Following a 2-h infection, Caco-2 cells were fixed in 4% paraformaldehyde for 10 min at 37°C, washed with PBS three times, and blocked with 2% bovine serum albumin (BSA) in PBS for 60 min at room temperature. Cells were incubated with primary antibody (anti-claudin-1, catalog no. 71-7800, and anti-ZO1, catalog no. 339100; Invitrogen, USA), with final concentration of 1:1,000, overnight at 4°C, washed three times, and incubated with secondary antibody and DAPI (4′,6-diamidino-2-phenylindole) for 1 h at room temperature in dark conditions. The samples were washed three times with PBS and mounted, and images were acquired using a confocal microscope (Nikon Eclipse Ti2; Japan) and analyzed using ImageJ. Scale bar, 20 μm. (B) Quantification of claudin-1 fluorescence. The error bars denote ± SEM. One-way analysis of variance (ANOVA) followed by Dunnett’s posttest was applied. Exact *P* values are provided. (C) Model of QS network in intestinal inflammation and permeability. Increased intestinal inflammation is driven by MvfR (solid arrow). MvfR triggers tight junction disruption and intestinal permeability indirectly (dashed arrow) at least through the P. aeruginosa QS network, which consists of three interconnected QS regulators, MvfR, LasR, and RhlR (color-coded interconnecting lines). Specifically, each regulator contributes to intestinal permeability, with MvfR functioning upstream of LasR and RhlR through a series of negative interactions (solid stop arrow), Solid stop arrow indicates inhibition. The specific nature of the regulatory relationships and feedback between these regulators *in vivo* is currently unknown.

Multiprotein tight junction (TJ) complexes are key regulators of the intestinal barrier function. Claudins are critical modulators of intestinal barrier integrity and have previously been identified to be altered in MvfR-mediated intestinal barrier dysfunction (19, 23). To determine how TJs are altered in Caco-2 intestinal cells following infection with each QS mutant strain, we monitored the TJ protein, claudin-1, by immunofluorescence staining. This revealed that while cells infected with the Δ*mvfR* mutant strain retained staining of this protein, cells infected with WT or any other QS mutant had a marked decrease in staining for claudin-1 (Fig. 2A and B). For uninfected and Δ*mvfR*-infected cells, the staining for claudin-1 was organized at the periphery of the cells, with a more uniform localization at the sites of cell-cell interaction (Fig. 2A), indicating that TJ integrity remained intact. These findings suggest that the increased intestinal permeability observed in mice may be mediated by a disruption of the TJ of the intestinal epithelium.

Taken together, these results indicate that the different effects the QS mutants have on intestinal inflammation and permeability are independent of the ability of each strain to disseminate to the gut. Furthermore, while inflammation and increased permeability are both QS-mediated consequences of infection by P. aeruginosa, the regulation of these host responses by QS is distinct (Fig. 2C). These findings provide important insights for predicting how mutations or inhibition of each QS system might impact the efficacy of anti-QS therapies. For instance, while inhibition of MvfR through genetic disruption (19) or with a non-ligand-based inhibitor (23) can prevent MvfR-driven increases in gut permeability, mutations in *lasR*, which is a common pathoadaptation in P. aeruginosa chronic infections, may impact the efficacy of an anti-MvfR approach to limiting gut permeability. These results also indicate that inhibitors targeting LasR or RhlR would likely have little effect on intestinal response to P. aeruginosa infection, and targeting RhlR and LasR may even worsen outcomes.

### Bacterial strains and growth conditions.

UCBPP-P. aeruginosa14 (PA14) is a rifampin-resistant P. aeruginosa human clinical isolate (Rahme laboratory) (25). Mutants are isogenic to UCBPP-PA14, *mvfR* (Rahme laboratory). The remaining single, double, and triple mutants were provided (Hogan laboratory) (7). Bacteria were grown in lysogeny broth (LB) agar plates. For each assay, overnight cultures were established from a single colony. Overnight cultures were then diluted 1:1,000 for a day culture grown at 37°C until an optical density at 600 nm (OD_600_) of 2.8 for the *in vivo* or an OD_600_ of 3.0 *in vitro* infection was reached.

### Infection studies in mice.

Experiments were performed with 10-week-old male C57BL/6 mice from Jackson Laboratories. Mice were maintained in a specific-pathogen-free environment at Massachusetts General Hospital (MGH; Boston, USA), in a 12-h-light/12-h-dark photoperiod at an ambient temperature of 22 ± 1°C, with food and water access *ad libitum*. Dorsal burn injury, 30% total body surface area (TBSA), and infection with approximately 3 × 10^5^ CFU of P. aeruginosa, tissue harvesting, and CFU counts on LB with 100-μg/mL rifampin plates were performed as previously described (23). The TNF-α and FITC dextran levels were detected as described previously (23).

Animal protocols were reviewed and approved by the Institutional Animal Care and Use Committee (IACUC) at MGH (protocol no. 2006N000093) and are in strict accordance with the guidelines of the Committee on Animals of the MGH, Harvard Medical School (Boston, USA), and the regulations of the Subcommittee on Research Animal Care of the MGH and the National Institutes of Health. Animals were euthanized according to the guidelines of the Animal Veterinary Medical Association. All efforts were made to minimize suffering.

### Cell culture studies.

Caco-2 cells (catalog no. HTB-37; ATCC) were grown on the removable chamber slide (catalog no. 80381, ibidi, USA) using Eagle’s minimal essential medium (EMEM) containing 10% heat-inactivated fetal bovine serum (FBS), 2 mM glutamine, and antibiotic-antimycotic at 37°C and 5% CO_2_, and cells were allowed to grow until they reached 100% confluence. Cells were washed three times with phosphate-buffered saline (PBS) and grown in antibiotic/antimycotic-free media for 3 h before infection. Cells were infected with bacterial cells at a multiplicity of infection (MOI) of 10 for 2 h. Cells were fixed, stained, and imaged as described in Fig. 2A.
